# Multimodal surface-based morphometry reveals diffuse cortical atrophy in traumatic brain injury.

**DOI:** 10.1186/1471-2342-9-20

**Published:** 2009-12-31

**Authors:** And U Turken, Timothy J Herron, Xiaojian Kang, Larry E O'Connor, Donna J Sorenson, Juliana V Baldo, David L Woods

**Affiliations:** 1 Veterans Affairs Northern California Health Care System, Martinez, CA, USA; 2 Department of Neurology, University of California, Davis, CA, USA

## Abstract

**Background:**

Patients with traumatic brain injury (TBI) often present with significant cognitive deficits without corresponding evidence of cortical damage on neuroradiological examinations. One explanation for this puzzling observation is that the diffuse cortical abnormalities that characterize TBI are difficult to detect with standard imaging procedures. Here we investigated a patient with severe TBI-related cognitive impairments whose scan was interpreted as normal by a board-certified radiologist in order to determine if quantitative neuroimaging could detect cortical abnormalities not evident with standard neuroimaging procedures.

**Methods:**

Cortical abnormalities were quantified using multimodal surfaced-based morphometry (MSBM) that statistically combined information from high-resolution structural MRI and diffusion tensor imaging (DTI). Normal values of cortical anatomy and cortical and pericortical DTI properties were quantified in a population of 43 healthy control subjects. Corresponding measures from the patient were obtained in two independent imaging sessions. These data were quantified using both the average values for each lobe and the measurements from each point on the cortical surface. The results were statistically analyzed as z-scores from the mean with a p < 0.05 criterion, corrected for multiple comparisons. False positive rates were verified by comparing the data from each control subject with the data from the remaining control population using identical statistical procedures.

**Results:**

The TBI patient showed significant regional abnormalities in cortical thickness, gray matter diffusivity and pericortical white matter integrity that replicated across imaging sessions. Consistent with the patient's impaired performance on neuropsychological tests of executive function, cortical abnormalities were most pronounced in the frontal lobes.

**Conclusions:**

MSBM is a promising tool for detecting subtle cortical abnormalities with high sensitivity and selectivity. MSBM may be particularly useful in evaluating cortical structure in TBI and other neurological conditions that produce diffuse abnormalities in both cortical structure and tissue properties.

## Background

Many TBI patients fail to show detectible abnormalities in cortical structure on standard neuroradiological examinations despite significant cognitive impairments [1-8]. In contrast, neuroimaging studies of TBI patient groups find highly consistent evidence of cortical gray matter atrophy [9-12]. Cortical atrophy is also found consistently in post-mortem investigations of TBI patients [13,14]. Diffuse axonal injury (DAI) and atrophy at the gray matter/white matter junction have been shown in post-mortem studies and in animal models of TBI [14-17].

The detection of cortical abnormalities in individual patients is essential for the accurate diagnosis and treatment of TBI patients. However, the variability in normal cortical anatomy makes it difficult to assess subtle alterations of cortical tissue properties. The variations in cortical folding patterns [18] and interregional differences in cortical tissue properties and gray matter thickness [19] necessitate methods that precisely align homologous regions of the cortical surface in different subjects into a standard coordinate frame using surface-based morphometric (SBM) analysis techniques [18-22]. SBM has permitted the detection of regional reductions in cortical thickness and folding in a variety of neurodegenerative and neuropsychiatric conditions [8,23-28].

While the application of SBM has previously been limited to the analysis of high-resolution T1-weighted anatomical images, we introduce here multimodal SBM (MSBM) that combines information from DTI and T1 image datasets. DTI-derived measures of tissue anisotropy and diffusivity [29,30] can be quantified in the cortex and in the underlying white matter [31-34]. Damage to cortical gray matter produces microstructural alterations in neuropil density, along with corresponding loss of projections in subjacent white matter fiber tracts. These changes are respectively associated with increased mean diffusivity (MD) and reduced fractional anisotropy (FA) [30,35,36]. While DTI has been used successfully to visualize deep white matter abnormalities in individual TBI patients [37-39], it has not yet been utilized to evaluate in individual patients the pericortical abnormalities that are suggested by animal models and post-mortem studies of TBI [13,16,40].

Multi-modal imaging can potentially enhance sensitivity to cortical abnormalities in individual cases. In theory, damage to neuronal cell bodies and axons should produce co-localized changes in cortical thickness (seen with high-resolution T1-weighted imaging), and neuropil density and fiber microarchitecture (seen with DTI). In addition, because neuronal death causes anterograde loss of axons, and axonal damage produces retrograde degeneration of neuronal cell bodies and dendrites [41,42], pericortical damage should produce a characteristic co-localized triad of abnormalities: reduced cortical thickness, increased cortical gray matter diffusivity, and reduced pericortical anisotropy. The detection of pathological tissue can therefore be enhanced with statistical procedures that combine evidence of co-localized abnormalities from multiple metrics of tissue integrity [43].

Here, we demonstrate the use of MSBM to characterize cortical abnormalities in a patient who suffered a severe TBI. MSBM revealed reliable and consistent regional cortical abnormalities that were not evident on a standard MRI evaluation.

## Methods

### Case presentation

**Figure 1:**
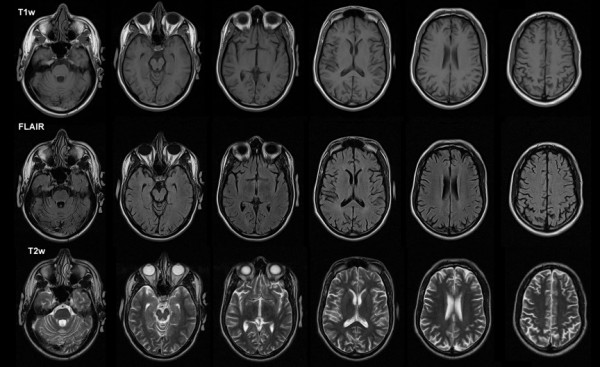
**Representative axial slices from clinical MRI scans of the TBI patient**. T1-weighted (top row), FLAIR (middle row) and T2-weighted images. The scans were interpreted as normal at the initial neuroradiological examination. Images are displayed according to radiological convention (right side of the brain is shown on the left side of figure).

A 33-year old right-handed male TBI patient with severe cognitive impairments was tested in 2008, 5 years post-injury. Following a motor vehicle accident in 2003, he remained in a coma for two weeks, with a Glasgow Coma Score (GCS) of 3 at hospital admission and a post-traumatic amnesia (PTA) lasting three weeks. A left fronto-temporal epidural hematoma and parieto-temporal skull fractures were observed on an initial computed tomography (CT) scan, but had resolved two years later on a repeat CT scan. His clinical MRI scans acquired three years post-injury (T1 with and without contrast, T2 and FLAIR) were reported as completely normal on an initial neuroradiological assessment (see Figure 1). As part of this research project, a retrospective assessment of these scans was performed by one of the authors (L.O.) who noted subtle cortical atrophy, and a small left orbito-frontal encephalomalacia that had not been detected in the initial examination. The patient has not been able to return to work due to chronic cognitive and emotional impairments. At the time of the brain imaging and the neuropsychological assessments reported here, the patient was on anti-depressant and mood stabilizing medications.

### Neuropsychological assessment

The patient was administered a comprehensive neuropsychological battery in January 2006, and showed significant impairment on tests of executive function, attention and memory. He was severely impaired on the visual scanning and number sequencing trials of the Delis-Kaplan Executive Function Scale (DKEFS) Trail-Making test (1^st ^and 2^nd ^percentile, respectively), though he performed somewhat better on the letter sequencing trial (9^th ^percentile, borderline impaired). On the DKEFS Color-Word Test, the patient was impaired across all trials (all 1^st ^percentile). The patient also performed poorly on a verbal working memory task, Wechsler Memory Scale (WMS)-III digit span, with scores in the 5^th ^percentile. He performed much better on the WMS-III spatial span task: 25^th ^percentile for forward span and 9^th ^percentile for backward span. He was also impaired on recalling the stories on the Logical Memory subtest of the WMS-III (1^st ^percentile). Memory impairments were also observed on the California Verbal Learning Test (CVLT)-II [44]. The patient was at the 1^st ^percentile for free recall on the five learning trials. He was also impaired on short-delay free recall (2nd percentile), long-delay free recall (7^th ^percentile), and recognition (13th percentile). However, on the long-delay forced-choice recognition portion of the CVLT-II, a measure of motivation/malingering, he was 100% accurate, thus ruling out poor motivation as a source of his impaired performance.

Importantly, the patient tested in the average range on a number of tests that do not rely on attention or memory. He performed well on the Wechsler Adult Intelligence Scale (WAIS)-III comprehension/knowledge subtest (63rd percentile) and on the DKEFS 20 Questions Test (42nd percentile), a test of reasoning and abstraction. He was in the normal range in his ability to copy the Rey-Osterreith Figure, a test of visuospatial perception, and on the Boston Naming Test, an index of language functioning. Last, he performed in the average range on the motor speed trial of the DKEFS Trail Test (37th percentile).

### Brain imaging

High-resolution T1-weighted structural and diffusion MRI scans were obtained using a 1.5T Phillips Eclipse scanner. To evaluate the reliability of the MSBM analysis, identical imaging sequences were acquired on two separate sessions at an interval of 6 days. Data from a third imaging session were discarded due to excessive patient motion during the DTI scans. T1-weighted images were acquired with a Spoiled Gradient Recall (SPGR) sequence (TR/TE = 15/4.47 ms, FOV = 240 mm, 256 × 256 imaging matrix, flip angle = 35°, 0.94 × 1.3 × 0.94 mm^3 ^voxels, 212 coronal slices). Two T1-weighted images were acquired and averaged together from each imaging session to improve signal-to-noise ratio (SNR). Two series of cardiac-gated diffusion images (single-shot spin echo EPI sequence, TR/TE = 600-1000/115.6 ms, FOV = 240 mm, 80 × 80 imaging matrix, flip angle = 90°, 3 × 3 × 3 mm^3 ^voxels, 48 axial slices, b = 1000 s/mm^2^) were acquired on each day of imaging using six non-collinear gradient directions.

### Control group

Normative imaging data were acquired from 43 young healthy volunteers (30 males, 13 females, mean age = 27, range = 18-48, all right-handed by self report) from the local community (San Francisco Bay Area, CA, USA).

### Informed consent

In accordance with the Declaration of Helsinki [45], written informed consent was obtained from the patient and all participants following protocols reviewed and approved by the VA Northern California Health Care System Institutional Review Board.

### Image post-processing

T1 images were corrected for magnetic field inhomogeneities and resampled to 1 × 1 × 1 mm^3 ^resolution. In order to correct for movement artifacts and geometric distortions [46], diffusion MRI images were co-registered with anatomical scans and resampled at the same resolution using SPM5 http://www.fil.ion.ucl.ac.uk/spm. MATLAB http://www.mathworks.com programs were implemented for processing the diffusion imaging data. A 2D 3.75 FWHM Gaussian smoothing filter was applied to individual slices from B0 images to correct for in-plane Gibbs ringing artifacts. An edge-preserving weighted-median tensor smoothing filter [47] was applied to the diffusion-weighted images for noise removal. The two DTI series from each imaging session were averaged to improve SNR. The diffusion tensor [30] was computed using log linear least squares while correcting for negative eigenvalues [48]. FA and MD maps [30] were then derived from the diffusion tensor.

### Surface-based morphometry

**Figure 2:**
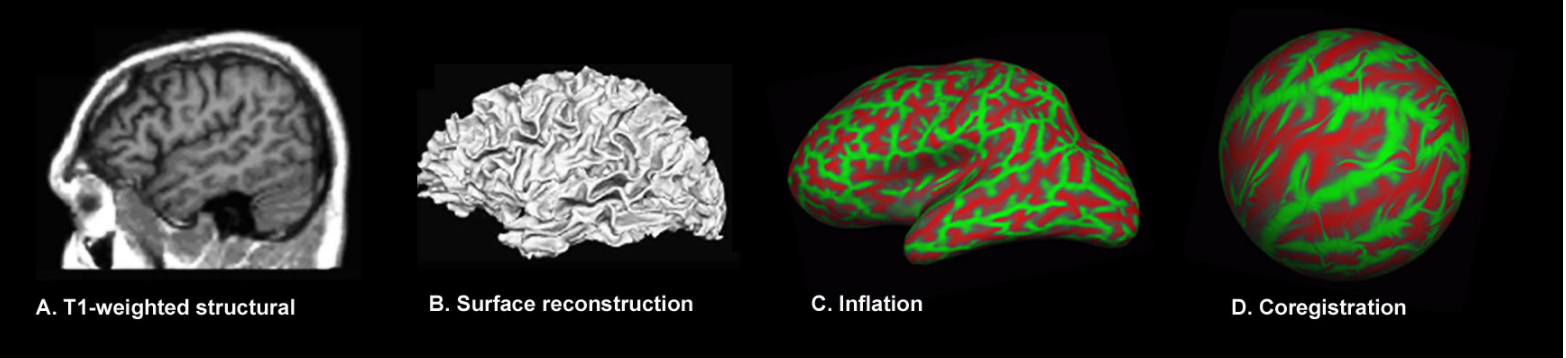
**SBM image processing steps: Surface reconstruction and alignment to standard template**. A. High-resolution T1-weighted images were processed using FreeSurfer 3.0 to map the cortical surface. The image is a lateral view of the TBI patient's left hemisphere. B. Computerized reconstruction of the gray/white matter boundary. A smoothed and expanded view of the white matter surface is shown. The image has been intensity normalized, skull-stripped and the cerebellum has been removed. C. Inflation of the cortical surface to map gyral and sulcal anatomy. Gyral regions are shown in green and sulcal regions in red. D. Coregistration of the subject's cortical surface to a common spherical template. This step allows the assessment of cortical tissue properties with respect to a normative database using a common coordinate system.

The cortical surface was reconstructed from high-resolution T1 images using the fully automated analysis protocol implemented in FreeSurfer 4.0 http://surfer.nmr.mgh.harvard.edu. First, the cortical surface was automatically segmented from the anatomical images (Figure 2A-C). Gyral anatomy was aligned to a standard spherical template using surface convexity and curvature measures (Figure 2D) [20,49,50]. Identical procedures were used to analyze control data to permit statistical comparisons of homologous cortical regions between the patient and controls. The frontal, temporal, parietal and occipital cortices delineated by the surface parcellation provided by FreeSurfer [51] were used to analyze cortical tissue properties at the lobar level.

**Figure 3:**
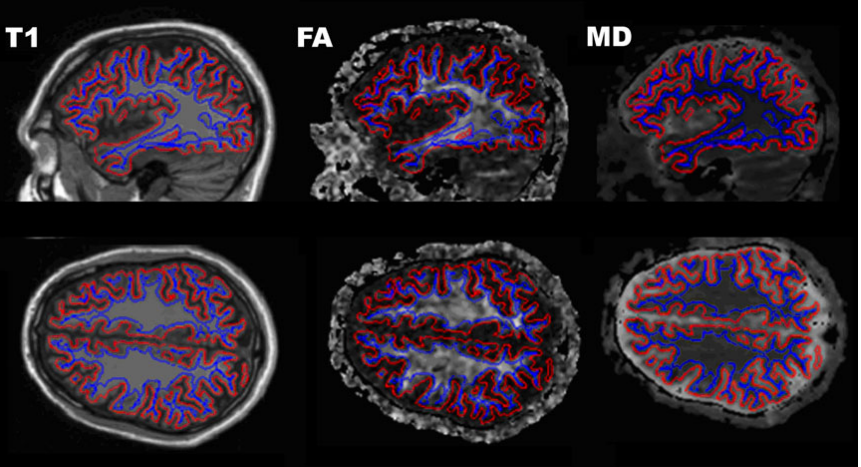
**Quantification of tissue properties in the cerebral cortex and in pericortical white matter**. Two surfaces were defined from the T1-weighted anatomical images (left column): in mid-gray matter (red) and 2 mm below the gray-white matter boundary (blue). White matter fractional anisotropy (middle) was quantified 2-mm below the gray/white boundary and mean diffusivity (right) was quantified in both the cortical gray matter and the white matter surfaces. Parasagittal (left hemisphere) and axial (above the lateral ventricles) cross-sections from the TBI patient's first imaging session are shown. High intensity regions on the FA map correspond to major fiber bundles running parallel to the cortical surface. Cerebrospinal fluid surrounding the cortex appears bright on the MD map.

FA and MD maps were resampled in mid-gray matter and in pericortical white matter 2 mm below the boundary between gray and white matter [34] (Figure 3). Cortical thickness [19] and DTI metrics were estimated at each point on a 1 mm^2 ^grid. In order to the minimize variance due to minor intersubject disagreements in gyral and sulcal anatomy, surface-based smoothing was implemented with a 2D 30 mm^2^ Gaussian kernel [52,53].

### Control data

**Figure 4:**
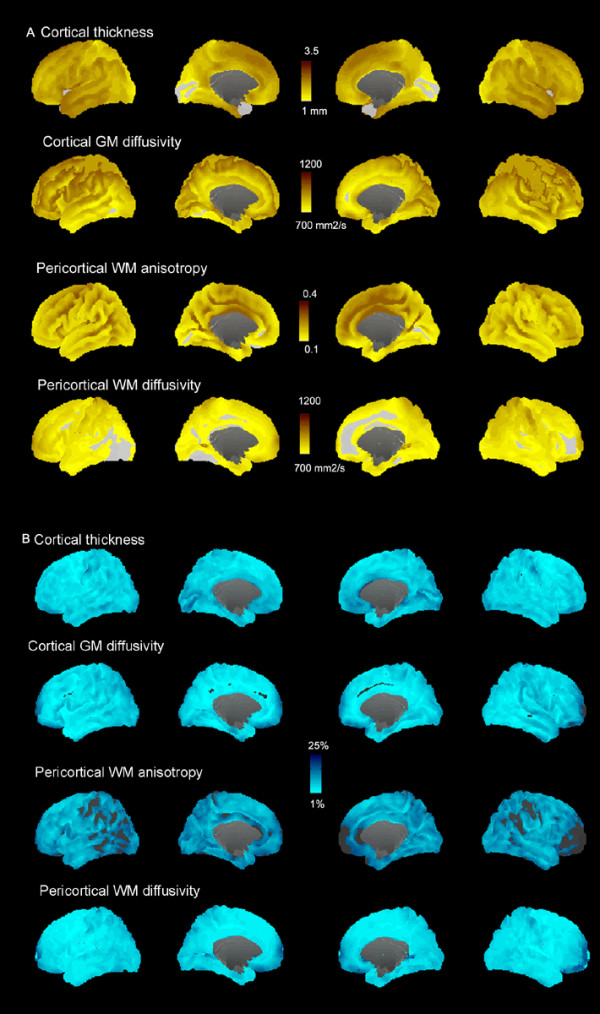
**A, B. Cortical gray matter and pericortical tissue properties quantified along the cortical mantle in control subjects**. Mean values (A) and variability (B, coefficient of variation) for cortical thickness, cortical mean diffusivity, and fractional anisotropy and mean diffusivity of pericortical white matter (2 mm below the gray-white boundary) for control subjects are shown for each point on the surfaces of the two hemispheres. The corpus callosum is cut out, and the regions with very low values (e.g., thickness < 1 mm, coefficient of variation < 1%) appear in gray.

Surface maps of mean cortical thickness, cortical diffusivity, pericortical white matter diffusivity and fractional anisotropy as well as their coefficients of variation (standard deviation/mean value) are shown for the control group in Figure 4. Average tissue properties of the different cortical lobes, together with their standard deviations, are shown in Table 1.

**Table 1:** Mean cortical tissue parameters in different lobes for the control group.

*Lobe*	Frontal		Occipital		Parietal		Temporal	
***Hemisphere***	**Left**	**Right**	**Left**	**Right**	**Left**	**Right**	**Left**	**Right**

Cortical GM thickness (mm)	2.68 ± 3.8%	2.67 ± 3.8%	2.03 ± 5.2%	2.04 ± 5.6%	2.34 ± 5.2%	2.33 ± 5.1%	2.88 ± 4.0%	2.90 ± 4.4%

Cortical GM diffusivity (mm^2^/s/1000)	1.05 ± 5.2%	1.03 ± 5.1%	0.97 ± 6.1%	1.02 ± 5.9%	1.06 ± 7.6%	1.09 ± 6.9%	0.93 ± 4.6%	0.95 ± 4.4%

Pericortical WM anisotropy	0.30 ± 4.3%	0.31 ± 4.5%	0.25 ± 9.7%	0.24 ± 6.3%	0.28 ± 8.4%	0.27 ± 5.3%	0.27 ± 7.2%	0.25 ± 5.6%

Pericortical WM diffusivity (mm^2^/s/1000)	0.85 ± 3.0%	0.83 ± 2.8%	0.83 ± 6.1%	0.87 ± 4.3%	0.85 ± 5.3%	0.90 ± 4.2%	0.83 ± 3.1%	0.84 ± 2.6%

Mean values and standard deviations (expressed as percentages) of cortical thickness, cortical mean diffusivity, and fractional anisotropy and mean diffusivity of pericortical white matter (2 mm below the gray-white boundary) for control subjects.

### Statistical procedures

Cortical tissue metrics from the patient were compared with those from control subjects at each point on the cortical and pericortical surfaces using one-tailed significance tests for reduced cortical thickness, lower pericortical anisotropy, and increased cortical and pericortical diffusivity. A significance threshold of *p *< 0.05, corrected for multiple comparisons, was achieved by setting the point-wise *z*-score threshold to 1.5 and by computing a variable cluster extent threshold based on parameter smoothness estimates [53,54]. To improve sensitivity, corrections for age, brain volume and local curvature were applied to the cortical thickness measure of control subjects [19,55]. In order to ensure the normal distributions of *z*-scores, transformations were empirically derived from the database and were applied to the thickness, FA, and MD parameter distributions separately. Because of greater statistical power in averaging over large cortical areas, z-scores were used to analyze data on the lobar level.

### Combined assessment of individual tests of each metric

The joint significance of the abnormalities on individual measures was assessed using a modified version of Fisher's combined probability test [56]. The original test uses the *p*-values (*p*_*i*_) from k independent single-sided tests sharing the same null hypothesis to calculate a joint test statistic: . The overall *p*-value can be computed from this test statistic using a χ^2 ^distribution with 2 k degrees of freedom. With covarying measures (e.g., cortical thickness and gray matter diffusivity) correlations between measurements from the normal group must be used to appropriately adjust the degrees of freedom of the reference χ^2 ^distribution [43,57]. The false-positive rate for the combined Fisher test was evaluated using leave-one-out permutation testing by comparing each control subject against the remaining control population using the same procedures as those used to analyze the patient data.

### Assessment of cortical surface alignment

**Figure 5:**
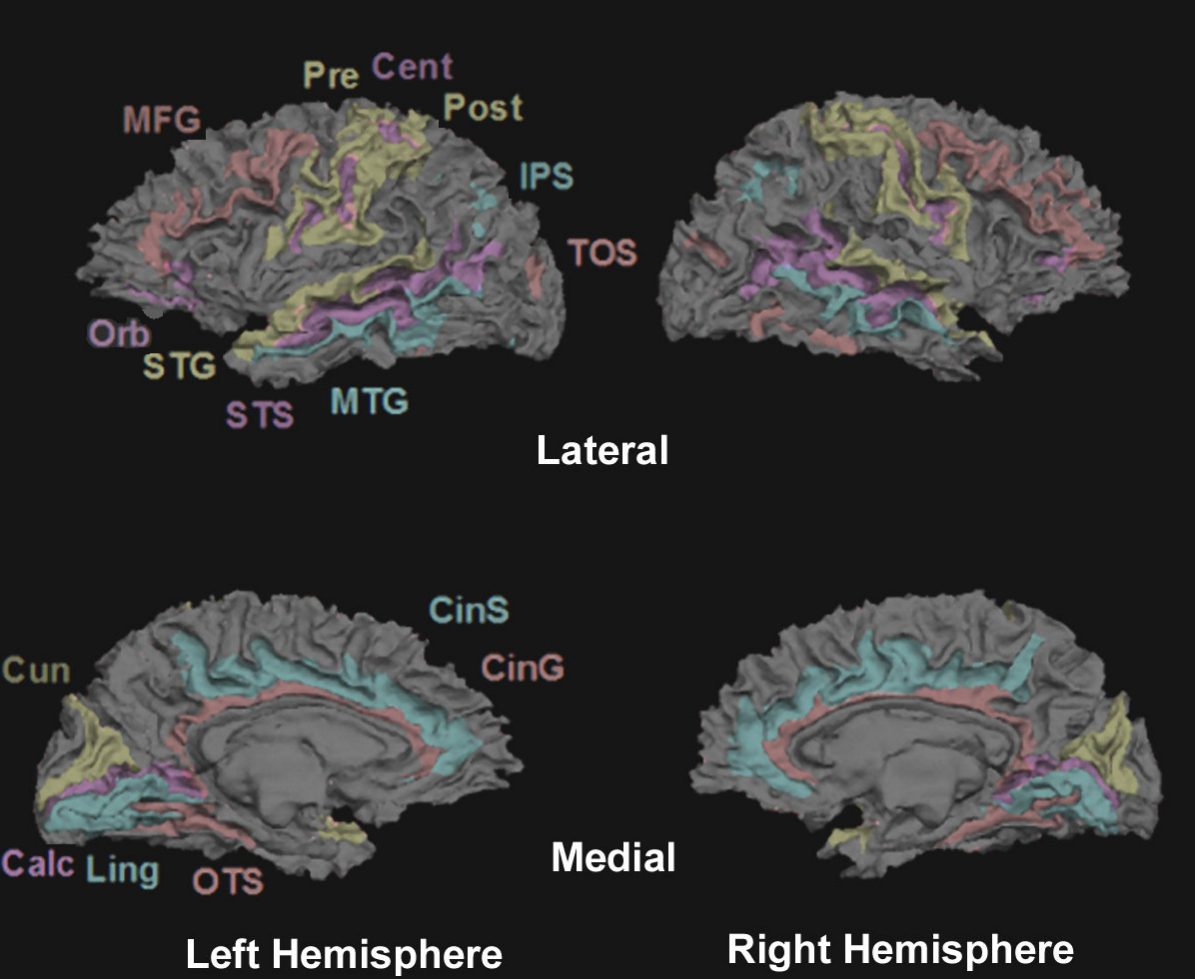
**Co-registration of the TBI patient's cortical surface anatomy with the Freesurfer atlas**. An accurate parcellation of the cortical surface was produced by Freesurfer shown superimposed on the semi-inflated surface of the patient (sulci = dark, gyri light). Cortical regions are accurately labeled. Anatomical labels: *Calc*, calcarine sulcus; *Cent*, central sulcus; *CinG*, cingulate gyrus; *CinS*, cingulate sulcus; *Cun*, cuneus; *IPS*, interparietal sulcus; *Ling*, lingual gyrus; *MFG*, medial frontal gyrus; *MTG*, medial temporal gyrus; *Pre*, precentral gyrus; *Post*, postcentral gyrus; *Orb*, orbital sulcus; *OTS*, occipito-temporal sulcus; *STG*, superior temporal gyrus; *STS*, superior temporal sulcus; *TOS*, transverse occipital sulcus.

Registration errors can occur when brain images from patients who have suffered brain atrophy are compared with images acquired from healthy controls, reducing the precision of structural comparisons. We assessed how well the cortical surface anatomy of the TBI patient was aligned with the mean cortical surface for the control group by visualizing major features of sulcal anatomy in relation to the atlas for the normal brain. Figure 5 shows the anatomical labels attached to selected gyri and sulci in the patient's brain by Freesurfer. Major gyral and sulcal structures were accurately identified and located close to population-average locations.

In order to assess alignment of surface anatomy and minor differences in sulcal structure at a finer scale (e.g. sulcal interruptions), we defined a curvature similarity metric at each location on the cortical surface. Surface curvature maps for each subject were compared with the mean curvature for the database, and the squared difference of a subject's regional cortical surface curvature from the mean was divided by the standard deviation of the database curvature to obtain a z-score curvature-similarity metric.

## Results

**Figure 6:**
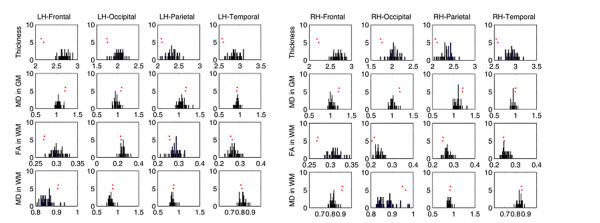
**Histograms showing the lobar measurements from the patient in relation to the distributions for the control group**. The distribution of cortical thickness, cortical mean diffusivity, and fractional anisotropy and mean diffusivity of pericortical white matter across 43 control subjects are shown for each lobe. The red dots indicate the corresponding measures from the patient's two imaging sessions.

Lobar analysis revealed significant bilateral cortical thinning in the TBI patient's frontal, temporal and occipital lobes (see Table 2, Figure 6). The frontal lobes showed the largest mean decrease in cortical thickness (left = -20.4%, right = 18.3%, p < 0.001). These were accompanied by significant increases in gray matter diffusivity (left = +15.6%, right = +17.4%, p < 0.001) and reductions in white matter anisotropy (left = -10.0% and right = -12.8%, p < 0.05). The joint assessment of all four metrics using the Fisher combined probability test detected highly significant abnormalities in the frontal lobes bilaterally (p < 0.001) as well as the right occipital lobe (p < 0.01) on both imaging sessions (Table 2, final two rows).

**Table 2:** Lobar-level analysis of cortical tissue integrity.

*Lobe*	Frontal		Occipital		Parietal		Temporal	
***Hemisphere***	**Left**	**Right**	**Left**	**Right**	**Left**	**Right**	**Left**	**Right**

Cortical GM Thickness	-20.4% 5.4***	-18.3% 4.7***	-14.1% 2.7*	-15.4% 2.7*	-10.7% **2.1**	-11.3% **2.2**	-10.5% 2.6*	-12.3% 2.8*

Cortical GM diffusivity	+15.6% 3.0*	+17.4% 3.4**	+13.1% **2.2**	+15.9% 2.7*	+10.4% **1.3**	+11.0% **1.6**	+0.6% **0.1**	+5.8% **1.3**

Pericortical WM anisotropy	-10.0% **2.3**	-12.8% 2.8*	-9.9% **1.0**	-9.3% **1.5**	-9.9% **1.2**	-7.9% **1.5**	-4.9% **0.7**	-4.0% **0.7**

Pericortical WM diffusivity	+6.5% **2.2**	+10.6% 3.8**	+6.5% **1.1**	+8.6% **2.0**	+3.5% **0.7**	+2.6% **0.6**	-2.0% **-0.6**	+1.8% **0.7**

Joint abnormality ( Fisher's z)	4.98***	5.54***	**2.27**	2.89*	**1.63**	**1.94**	**1.34**	**2.20**

Joint abnormality Replication	4.58***	5.22***	**2.02**	3.18*	**1.45**	**1.74**	**1.04**	**1.94**

Percentage difference in the TBI patient with respect to mean values for the control group and the corresponding z-scores (boldface) are shown for cortical thickness, cortical mean diffusivity, and fractional anisotropy and mean diffusivity of pericortical white matter (2 mm below the gray-white boundary). Results are from the first imaging session. Asterisks (*, **, ***) indicate z-scores significant at the *p *< 0.05, 0.005, 0.0005 levels (*z *> 2.5, 3.3, 3.9, respectively), Bonferroni corrected for multiple comparisons across the four lobes and the two hemispheres. The bottom two rows show joint z-scores of all tissue metrics using Fisher's combined test for imaging sessions 1 and session 2.

**Figure 7:**
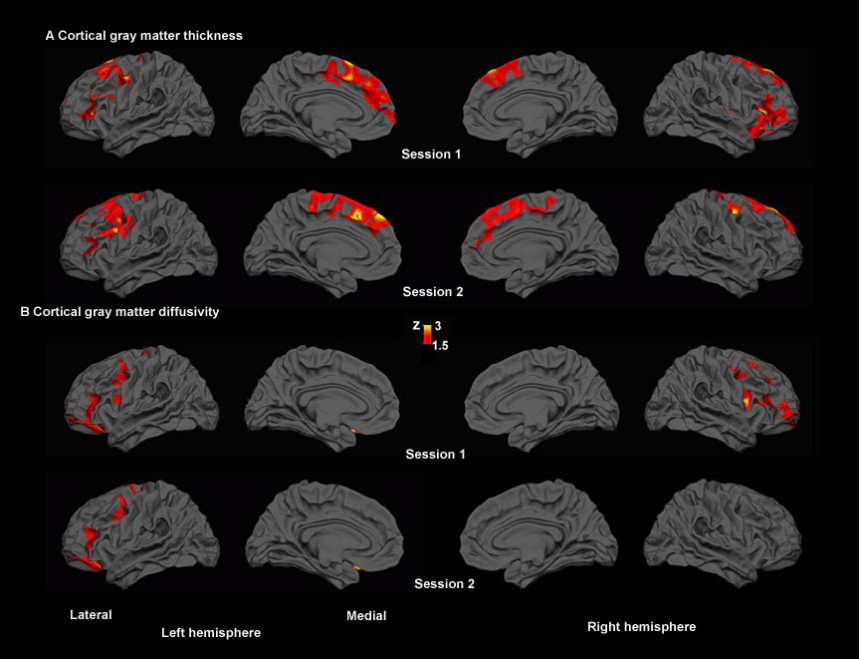
**Regional cortical gray matter abnormalities detected by SBM in the TBI patient**. Reduced cortical thickness (A) and increased cortical gray matter diffusivity (B) were most pronounced in the frontal lobes in two imaging sessions. Each row shows lateral and medial inflated views of the two hemispheres. Color bar shows z-values.

**Figure 8:**
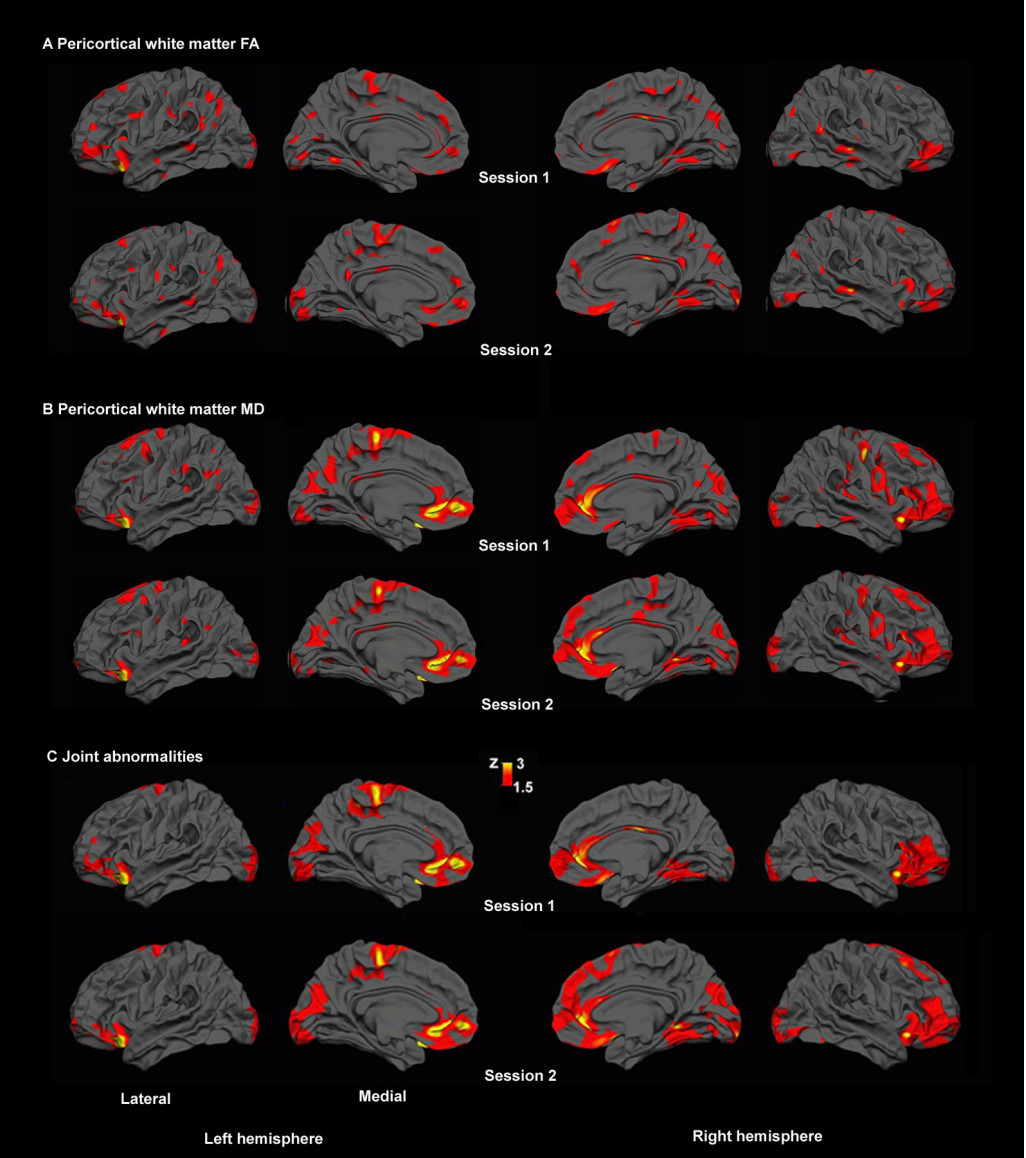
**Pericortical white matter abnormalities**. Regions of low anisotropy (A) and high diffusivity (B) were broadly distributed underneath the cortical mantle, with similar spatial distributions in both sessions (point-wise, z > 1.5, shown without the cluster threshold).

MSBM surface analysis revealed focal regions of abnormally thin cortex in the lateral and medial frontal cortex (see Figure 7A). There was also increased gray matter diffusivity in lateral frontal regions (Figure 7B). Analysis of pericortical white matter revealed broadly distributed regions of reduced anisotropy and increased diffusivity that replicated across two imaging sessions (Figure 8A, B).

**Figure 9:**
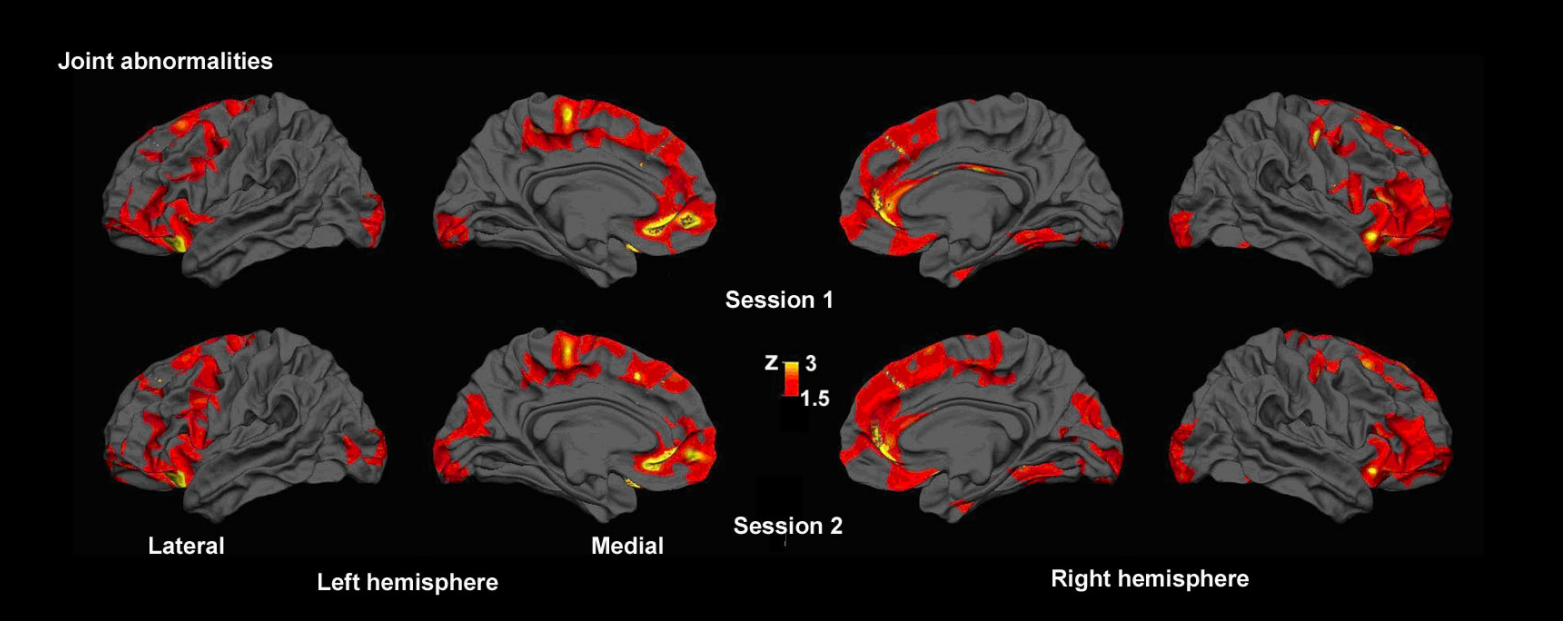
**Combined assessment of cortical abnormalities**. Joint tests of significant cortical gray matter and pericortical white matter abnormalities using Fisher's combined probability test revealed extensive abnormalities, concentrated mainly in the frontal lobes and basal occipito-temporal regions. The spatial distribution of the abnormalities replicated on the two different imaging sessions.

Combined assessments of all four metrics (cortical thickness, gray matter diffusivity, pericortical white matter anisotropy and diffusivity) showed extensive abnormalities in the frontal lobes and in basal occipito-temporal regions bilaterally. More spatially restricted abnormalities were also found in the inferior temporal lobe of the right hemisphere and the medial parietal regions and the posterior cingulate gyrus of the left hemisphere (Figure 9). The regions showing abnormalities were almost identical on the two imaging sessions. They were spread over 29.1% and 27.5% of the total surface area of the left hemisphere in the two imaging sessions, and over 31.4% and 31.5% of the right hemisphere.

No such widespread abnormalities were detected in any of the control subjects. The leave-one-out procedure using Fisher's combined probability test found statistical abnormalities in four of the 86 normal control hemispheres, resulting in an overall 4.7% false positive rate. Somewhat higher false-positive rates were seen for individual measures: 12.8% for thickness and 8.1% for other measures. Among the control population, one control subject had bilateral abnormalities, and two others had abnormalities restricted to a single hemisphere. In these control subjects, the extent of the statistically significant joint abnormalities was 1.6%, 4.6%, 5.1%, and 6.6% of the total surface area in the four hemispheres that showed statistical abnormalities.

Assessment of the cortical surface curvature similarity between the patient and controls revealed marginally significant differences in fine cortical structure in the left frontal, left parietal and bilateral temporal lobes on the first scanning session. Significant differences were restricted to the left temporal lobe in the second session (Table 3). We therefore tested whether there was a systematic relationship between surface curvature and the abnormalities detected in the point-wise joint significance tests at each point on the cortical surface. Pearson correlation values for the first and second scanning sessions were 0.033 and 0.046, respectively, in the left hemisphere and were -0.020 and -0.059 in the right hemisphere. None of these effects reached statistical significance.

**Table 3:** Cortical surface curvature compared between the patient and the control group.

*Lobe*	Frontal		Occipital		Parietal		Temporal	
***Hemisphere***	**Left**	**Right**	**Left**	**Right**	**Left**	**Right**	**Left**	**Right**

Session 1	2.5*	1.9	-0.9	0.8	5.6*	1.7	3.0*	2.5*

Session 2	1.8	0.7	-1.2	0.1	2.3	2.3	3.5*	1.5

Z-scores indicate for each lobe the extent to which surface curvature differs between the patient and the control group. Results are from two separate imaging sessions. An asterisk (*) indicates a z-score significant at the p < 0.05 level, Bonferroni corrected across lobes and hemispheres. The results are presented by lobe for the two hemispheres.

## Discussion

We used multimodal surface-based morphometry (MSBM) to identify widespread cortical abnormalities in a TBI patient with minimal abnormalities evident on standard MR imaging. Specifically, we tested for reduced cortical thickness and pericortical white matter anisotropy as well as increased gray and white matter diffusivity in the patient relative to a control group. At the lobar level, region-of-interest analyses revealed extensive abnormalities in the frontal cortices and the underlying white matter. These abnormalities were consistent across imaging sessions. When examined with different metrics, abnormalities were typically localized to the same cortical regions, enhancing the statistical significance using Fisher's combined-metric analysis. The patient showed extensive bilateral abnormalities in frontal and basal occipito-temporal regions, with more focal abnormalities found in other locations (the left posterior cingulate cortex, the left medial parietal lobe and in the right inferior temporal lobe).

The control population showed predictably low false-positive rates (4.7%). Moreover, the false-positive abnormalities detected in the control population were much less extensive than those observed in the patient. Because identical statistical procedures were used to evaluate each control subject and the patient, this indicates that the abnormalities observed in the patient were far outside the normal range of variability.

The effects observed in this patient are consistent with the diffuse cortical atrophy that is observed consistently in animal models of head trauma [16,17], and neuroimaging investigations of TBI patient groups [9,10]. Increased cortical diffusivity is consistent with neuropil density reductions seen in histological investigations of TBI [42]. Pericortical white matter abnormalities are consistent with the vulnerability of the gray-white matter junction to diffuse axonal injury (DAI) [13,16]. The frontal and ventral distribution of the abnormalities is consistent with typical findings for TBI-related cortical damage [1,2,4,58,59].

The extensive frontal lobe abnormalities are consistent with the TBI patient's poor performance in neuropsychological tests of executive function and attention. The diffuse lateral prefrontal cortex plays an important role in cognitive control [60], and brain lesions in this region produce deficits in high-level cognitive abilities [61]. The extent of the lateral prefrontal abnormalities (Figure 7), including the left dorsolateral frontal cortex which is critical for cognitive control [62], can help explain the patient's severe and chronic cognitive impairments despite the absence of brain lesions evident in standard neuroradiological examination.

### Diagnostic value of surface-based morphometry

The cortical abnormalities revealed by MSBM are difficult to detect on visual inspection of standard MRI slices for several reasons. First, the abnormalities are small in magnitude but widespread, spanning multiple image cross-sections. In addition, they are difficult to detect because the thickness of the cortical ribbon seen on cross sections varies substantially because of the systematic gray matter thickness differences across cortical regions [19]. MSBM permits the automated, quantitative detection of abnormalities across the cortical surface that are otherwise difficult to detect on standard neuroradiological assessments.

### Assessment of cortical and pericortical tissue properties using DTI

Diffusion tensor imaging has been used successfully to characterize deep white matter abnormalities in TBI. With the present approach, white matter integrity adjacent to cortex can also be assessed. DAI has a predilection for the gray-white matter junction [4] in both animal models [16,40] and post-mortem human investigations [13]. In the current study, lobar analysis revealed widespread anisotropy reduction and diffusivity increase in frontal pericortical white matter. Point-wise analysis found multiple smaller patches of reduced anisotropy and increased diffusivity beneath the cortical mantle.

The architecture of white matter near the cortex is complex, with both short- and long-range fiber systems terminating in the same cortical zones [33,63]. Inspection of the FA map in Figure 3 reveals the large anisotropy variations along the cortical sheet, partly reflecting the manner in which the cortical surface makes contact with the deep white matter tracts. DTI measurements are also less reliable in or near the cortex than in deep white matter [64]. The development of improved image post-processing and quantification procedures for quantifying DTI metrics near the cortex [32-34,65] will further improve the assessment of pericortical white matter integrity.

### Joint MSBM assessment of tissue integrity

Co-localized abnormalities evident with different tissue metrics were identified by the combined analysis of multi-modal data (e.g., the right frontal and right occipital lobe abnormalities in Table 1). As expected, TBI-related neuropathological processes produced co-localized abnormalities, reflecting the causal link between the integrity of neuronal cell bodies, their dendrites, and their axons [41]. Subthreshold abnormalities that might be masked by high intersubject variability or measurement noise can be detected with higher sensitivity when assessed conjointly. For instance, the regionally-specific abnormalities in white matter anisotropy and diffusivity did not reach significance in isolation (Figure 8) but their joint assessment revealed significant white matter abnormalities consistent with DAI. Moreover, these abnormalities were typically found in regions with significant cortical thinning. In contrast, artifact-related abnormalities are unlikely to be aligned across imaging modalities. Thus, the Fisher combined probability test is a promising approach for reducing the likelihood of detecting artifactual differences while maximizing sensitivity [43,57].

### Image co-registration

When comparing morphometric data from patients who suffered brain atrophy with a healthy control group, it is important to consider the possibility of co-registration errors due to global shape changes as well as alterations in fine-level texture of brain tissue. While smoothing serves to compensate for registration errors, there is a corresponding loss of spatial localization accuracy and the potential for partial-voluming due to averaging over dissimilar structures. These sources of error need to be carefully assessed when interpreting the findings from individual patients.

In the present investigation, the TBI patient's brain anatomy was judged to be normal on his initial radiological investigation, and his cortical anatomy aligned successfully with normal brain templates. However, we found significant differences in fine-level cortical surface curvature measures in the patient and controls. This may have reflected changes in gyral morphology consequent to cortical tissue loss. However, the differences in curvature did not predict the regions showing significant tissue abnormalities. This likely reflects a difference in spatial scale of the two measures. Curvature differences largely reflect sub-centimeter alterations in gyral morphology, while surface-based analyses using a 30 mm smoothing filter reflect differences on a coarser spatial scale [52,53]. Cortical thickness and DTI-derived measures were also averaged over the four lobes of the two hemispheres, and significant bilateral abnormalities were detected in the patient's frontal lobes. The lobar-level analysis does not rely on the precise alignment of fine sulcal structure, and provides a way to assess tissue abnormalities that is robust with respect to potential image-coregistration problems in patient populations.

The analysis of imaging data from patients with both focal lesions and possibly subtle and diffuse tissue alterations presents a technical challenge for automated morphometric assessment methods. Image co-registration algorithms are liable to distortions due to the presence of such lesions [66]. While the effects of focal lesions on the performance of volume-based co-registration algorithms has been investigated [66-68], it will be important for future investigations to assess how surface-based registration is affected by the presence of lesions and how possible distortions can be addressed.

### Generalizability of the MSBM approach

The sensitivity of MSBM in detecting abnormalities in other TBI patients remains to be determined. TBI patients constitute a heterogeneous population, with varying etiology and neuropathology.

However, common patterns of cortical atrophy that are especially pronounced in frontal lobes have been reported consistently in imaging studies of TBI patient groups [9-12]. TBI is associated with significant reductions (up to 10%) in whole brain volume and total gray matter volume [69-71], with greater TBI severity associated with more gray matter loss. This suggests that cortical thickness reductions ranging from 0.15 to 0.3 mm can be expected in moderate to severe TBI cases. Our normative data (Table 1) indicate that a 10% decrease can be detected at the lobar level with the present approach. Further improvements in sensitivity to co-localized cortical gray matter and pericortical white matter abnormalities are achieved with multi-modal integration.

TBI-related damage to long white matter tracts has also been well documented [72-74] and is associated with reductions in white matter fractional anisotropy of up to 20%, and increases in mean diffusivity of up to 15% [75,76]. The lobar-level analyses presented here are sensitive to abnormalities in tissue properties (e.g., 15% in fractional anisotropy and 10% in mean diffusivity) that would fall in the mid-range of the TBI-related anisotropy and diffusivity alterations reported in other structures.

The combined analysis increases sensitivity to borderline abnormalities in moderate and severe TBI cases that present a quandary for radiological diagnosis. The normative data presented in Figure 4 and Table 1 indicate that the power to detect abnormalities is not uniform over the cortex in any of the imaged modalities. Further, the cortical regions with low coefficients of variation have different distributions for different modalities. Therefore, multi-modal assessment should improve the power to detect statistically significant abnormalities in any cortical region. Progressive improvement in the automated detection of abnormalities will also occur as more modalities are incorporated into the imaging protocols [77].

Other approaches based on multivariate classification have also been proposed to formally integrate multi-modal data in group studies to increase the power to detect disease-related abnormalities [78,79]. Our statistical approach relies on characterizing the normal variability in data from different imaging modalities, and combining the results from a series of statistical tests in relation to the covariance structure of the normative data. Each test can be interpreted alone (e.g., reduced cortical thickness and low anisotropy in TBI) and in combination. This approach can be easily adapted to incorporate a larger number of imaging modalities and generalized to patient populations with different etiologies that may produce diffuse alterations in cortical structure and microarchitecture.

## Conclusions

The present findings highlight the potential of multimodal surface-based morphometry (MSBM) for detecting and characterizing TBI-related diffuse cortical pathology. MSBM is a promising, generalizable method that permits the objective quantification and evaluation of subtle cortical abnormalities in TBI as well as other neurological, psychiatric and developmental conditions that produce diffuse alterations in cortical structure.

## Competing interests

The authors declare that they have no competing interests.

## Authors' contributions

Data collection, subject and patient recruitment: DLW, XK, AUT, LEO, JVB, DS; Diagnostic judgment: LEO, JVB; Data analysis: AUT, TJH, XK, DLW; Literature review: AUT, TJH, DLW; Theoretical interpretation: AUT, TJH, DLW; Preparation of drafts of the manuscript: AUT, DLW, TJH. All authors read and approved the final manuscript.

## Pre-publication history

The pre-publication history for this paper can be accessed here:

http://www.biomedcentral.com/1471-2342/9/20/prepub
